# Opportunistic pathogenic fungi isolated from feces of feral pigeons in Mafikeng, North West Province of South Africa

**DOI:** 10.14202/vetworld.2019.1066-1069

**Published:** 2019-07-18

**Authors:** Michelo Syakalima, Tsepo Ramatla, Ngoma Lubanza

**Affiliations:** Department of Animal Health, School of Agriculture, Faculty of Natural and Agricultural Sciences, Mafikeng Campus, North-West University, Private Bag X2046, Mmabatho 2735, South Africa

**Keywords:** chain reaction, fungi, opportunistic pathogens, pigeon feces, polymerase

## Abstract

**Background and Aim:**

Pigeon feces are increasingly being implicated in the spread of bacterial pathogens such as *Escherichia coli*, *Campylobacter*, *Salmonella*, *Listeria*, and *Chlamydia*. Fungi are rarely investigated except for *Cryptococcus* that has emerged as an important pathogen in old people and immunosuppressed patients. This study investigated fungi in pigeon feces collected from Mafikeng, the North West Province of South Africa.

**Materials and Methods:**

Freshly dropped feces were collected and enriched in phosphate-buffered saline overnight at 48°C and then subcultured on Sabouraud’s dextrose agar and incubated at 48°C for 2 weeks observing any fungal growth from day 2. The growths were picked up, DNA extracted, and polymerase chain reaction was done using the internal transcribed spacer primers.

**Results:**

Fungi isolated included: *Aspergillus* (*Aspergillus tubingensis*), *Cryptococcus* (*Cryptococcus albidus* and *Cryptococcus randhawai*), *Fusarium* spp., and *Rhodotorula* (*Rhodotorula mucilaginosa* and *Rhodotorula kratochvilovae*). Most of these isolates are known opportunistic pathogens and have been isolated in clinical conditions elsewhere. Other isolates such as *Graphium dubautiae*, *Myrmecridium schulzeri*, *Naganishia albida*, *Paecilomyces lilacinus*, and *Zygopleurage zygospora* were not found to be of any human health significance.

**Conclusion:**

We, therefore, concluded that the presence of these opportunistic pathogens is a significant human health risk, especially in the face of the HIV/AIDS pandemic that results in immunosuppression.

## Introduction

Feral pigeons frequently reside in close proximity to human habitats consequently dropping feces and littering the environment that they share with humans. Their presence in the human environment and the litter they produce is increasingly being recognized as a source of significant human pathogens responsible for some very important infections [1]. Pathogens such as *Escherichia coli*, *Campylobacter, Salmonella, Listeria, Chlamydia*, and *Cryptococcus* have been isolated from these pigeons and are known to be spread to humans through the air, food, or drinking water contaminated by pigeon droppings, nesting materials, or even dead carcasses [2]. Pigeons may also carry mites, which are vectors of pathogens that may find their way to humans and other animals.

Of the pathogens carried by pigeons, fungi are rarely investigated except for *Cryptococcus* that is well-known for fatal meningitis; it causes on immunosuppressed humans such as the old, sick, and HIV-positive patients. Other emerging opportunistic fungal infections from genera such as *Aspergillus, Penicillium, Mucor, Rhizopus, Paecilomyces, Fusarium, Alternaria*, and *Cladosporium* are gaining recognition and have been implicated in a variety of infections in humans [3-8]. Pigeons are known to carry fungi that are pathogenic to human such as those that cause Cryptococcal meningitis, because of this there is interest in knowing the pathogenic role of other fungi that may be found in pigeons.

Feral pigeons are an ever-increasing presence in the North West Province of South Africa; however, few investigations exist on their probable public health risk. This study investigated fungi in feces of pigeons collected around Mafikeng, the capital city of the North West Province of South Africa with special emphasis on fungi that are known to be opportunistic pathogens to humans.

## Materials and Methods

### Ethical approval

The fecal samples were collected where the pigeons nest. Therefore, the present study did not engage any invasive procedure and therefore, ethical approval was not required. Furthermore, the pigeons are not an endangered species and their feces are routinely cleaned and disposed off as a nuisance.

### Study site, sample collection, and processing

Fresh fecal samples of pigeons (*Columba livia*) were collected early in the morning from buildings around Mafikeng where the pigeons nest. Mafikeng is the capital of the North West Province of South Africa and is the hub of major agricultural activities. There was no requirement for permission to sample the feces because they were normally regarded as a nuisance and dirt in the area and the birds are not endangered species.

The freshly dropped feces were identified and a scoop of more than a gram of feces was done to the top layer of feces using sterile spatulas to minimize contamination. After collection, the feces were placed in screw-capped sterile sampling tubes and placed in a cooler box with ice packs for transportation to the laboratory at the North-West University, Mafikeng Campus.

At the laboratory, the fecal samples (1.0 g) were suspended in a 10.0 ml volume of sterilized phosphate-buffered saline pH 7.2. They were left in this solution overnight at 48°C. The next day 0.1 ml aliquot of each dilution was spread uniformly on Sabouraud’s dextrose agar (Sigma-Aldrich, South Africa) and the plates were incubated at 48°C for 2 weeks observing them for any fungal growth from day 2.

When the appearance of colonies was observed, the characteristics of these colonies were noted and some of the representative types of colonies were further subcultured and incubated in a similar manner above to get pure colonies. The pure colonies were then picked up and used for DNA extraction.

### Extraction of genomic DNA

For DNA genomic extraction, a single pure colony from the subcultures was picked up and subjected to a DNA extraction protocol of the Quick-DNA^™^ Fungal/Bacterial Miniprep Kit (Zymo Genomic DNA-Tissue MiniPrep, USA). The manufacturer’s recommendations were strictly adhered to. The DNA concentration was determined using NanoDrop ND-1000 ultraviolet (UV) spectrophotometer (Thermo-Fisher Scientific Inc., USA) with a wavelength of 260 nm. The resultant DNA eluted was then kept at −80°C until polymerase chain reaction (PCR) was performed.

### DNA amplification

The fungi isolates were identified by amplification of the internal transcribed spacer (ITS) gene. The ITS gene fragment is specific to fungi and most fungi yield products from 600 to 650 bp [9]. The primer set used to amplify the ITS region were ITS1 (5’-TCC GTA GGT GAA CCT GC GG-3’) and ITS4 (R-TCC TCC GCT TAT TGA TAT GC-3’). The PCR was done in a total volume of 25 μL of reaction mixture containing 1 μL of each primer pair (10 pM), 1 unit of Taq polymerase, 0.25 mM dNTP, 10 mM Tris-HCl (pH 9.0), 30 mM KCl, 1.5 mM MgCl_2_, and 1 μL of DNA template (1 μg/µL). PCR amplification was performed with a DNA thermal cycler (model – Bio-RAD C1000 Touch TM Thermal Cycler) using the following conditions: Initial denaturation at 94°C for 5 min; 35 cycles of 94°C for 1 min, 53°C for 1 min, and 72°C for 90 s, and a final extension at 72°C.

### Electrophoresis of PCR products

The PCR products were separated by electrophoresis on 1.5% (w/v) agarose gel on a horizontal Bio-Rad equipment system (model – BCMSCHOICE; Biocom, UK). The gel was run for 60 min at 80 V and 250 MA using 1×TAE buffer (40 mM Tris, 1 mM EDTA, and 40 mM glacial acetic acid, pH 8.0). Each gel contained a 1000 bp DNA molecular weight marker (Fermentas, USA). The gel was stained in ethidium bromide (0.1 μg/ml) and amplicons were visualized under UV light at 420 nm wavelength. The PCR product for sequencing was purified at Inqaba Biotech Laboratories in Pretoria, South Africa.

The resulting sequences were compared with reference data available in the GenBank database using BLAST (www.ncbi.nlm.nih.gov/BLAST) from the National Centre for Biotechnology Information (NCBI) to determine species identification with high similarity [10].

### Phylogenetic relationships

The evolutionary history was inferred using the maximum likelihood method based on the Jukes-Cantor model [11]. Initial tree(s) for the heuristic search were obtained automatically by applying neighbor-joining and BioNJ algorithms to a matrix of pairwise distances estimated using the maximum composite likelihood approach and then selecting the topology with superior log-likelihood value. The tree was drawn to scale, with branch lengths measured in the number of substitutions per site. The analysis involved 35 nucleotide sequences. Codon positions included were 1^st^+2^nd^+3^rd^+Non-coding. All positions containing gaps and missing data were eliminated. There were a total of 490 positions in the final dataset. Evolutionary analyses were conducted in MEGA7 software (Pennsylvania University, USA) [12].

## Results

**Figure-1:**
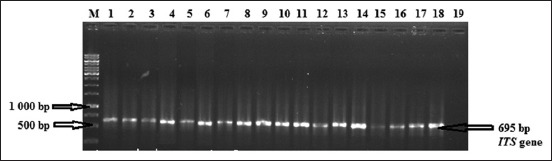
Electrophoresis in a 1.5% agarose gel of polymerase chain reaction amplified internal transcribed spacer (ITS) gene. Lane M: Molecular weight marker (1 kb); Lane 1-18 (ITS gene fragments from DNA extracted from pigeons feces), Line 19: Negative control.

For a total of 30 samples that were cultured on Sabouraud’s dextrose agar, 20 (66.6%) had colonies that gave positive reactions to the ITS primers with resulting bands on the gel that fell within the 600-650 bp range (Figure-1).

After sequencing and blasting on the NCBI database, the identities of the fungi were known as shown below together with their percentages similarities and accession numbers. The sequences obtained from the current study have been submitted to the GenBank database and assigned accession numbers are shown in Table-1.

**Table 1:** All the fungi types that were identified from the samples collected.

Samples ID	Reference from NCBI database	Percentage similarity (%)	Accession number in GenBank (PCR)	Assigned accession numbers
NWU 1	*Naganishia albida*	99	KY744108.1	MG551274
NWU 2	*Rhodotorula kratochvilovae*	99	KY104785.1	MG551275
NWU 3	*Myrmecridium schulzeri*	99	KP132479.1	MG551276
NWU 4	*Aspergillus tubingensis*	100	JF411067.1	MG551277
NWU 5	*Naganishia albida*	99	KC515372.1	MG551278
NWU 7	*Aspergillus tubingensis*	100	JF411067.1	MG551279
NWU 8	*Rhodotorula mucilaginosa*	100	KC816558.1	MG551280
NWU 9	*Microascus croci*	99	KX923854.1	MG551281
NWU 10	*Cryptococcus albidus*	99	KY445947.1	MG551282
NWU 11	*Aspergillus* sp.	100	HQ649946.1	MG551283
NWU 12	*Cryptococcus albidus*	100	HQ234288.1	MG551284
NWU 13	*Papiliotrema laurentii*	100	KY104470.1	MG551285
NWU 14	*Rhodotorula kratochvilovae*	100	KY104785.1	MG551286
NWU 16	*Naganishia albida*	99	MG250421.1	MG551287
NWU 17	*Naganishia albida*	100	KU883298.1	MG551288

PCR=Polymerase chain reaction, NCBI=National Center for Biotechnology Information

**Figure-2:**
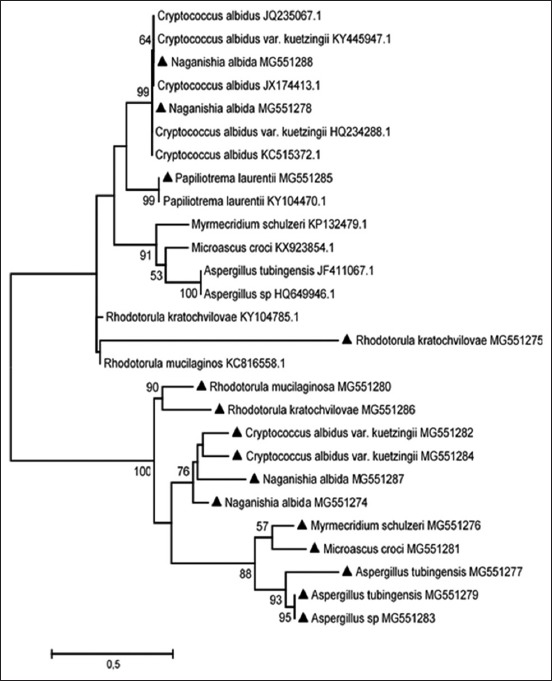
The evolutionary history of isolated fungi using the maximum likelihood method.

The tree with the highest log likelihood is shown in Figure-2 and represents similar fungi isolated in studies elsewhere, especially from infections. The percentage of likelihood, in which the associated taxa clustered together, is shown next to the branches.

## Discussion

This study isolated and characterized a number of fungi groups that are known to pose significant opportunistic risks. These included *Aspergillu*s, *Cryptococcus*, *Fusarium*, and *Rhodotorula* genera. The phylogenetic tree indicates some strains similar to the ones in this study that was isolated elsewhere and was involved in various opportunistic infections.

*Aspergillus* species are known to cause significant morbidity and mortality in humans. They are associated with a number of clinical forms such as disseminated infections, respiratory infections, subcutaneous infections, rhinocerebral infections, skin and nail infections, ear infections, and keratitis [12]. *Aspergillus* *tubingensis* was identified in the pigeon feces in this study and is a known opportunistic pathogen. *A. tubingensis* is most often involved in food spoilage of fruits and wheat, and industrial fermentation; however, it is also a rare agent of opportunistic infections such as corneal infections [13] as well as infections of maxillary bone following a tooth extraction [14]. Its presence in pigeon feces and contamination of the human environment should, therefore, raise public health concerns, especially for the elderly and immunosuppressed.

A number of *Cryptococcus* species were also isolated from these fecal samples and this genus, in general, is regarded a public health risk. Under this genus, *Cryptococcus neoformans* is the major human and animal pathogen responsible for serious disease in immunosuppressed patients. *C. neoformans* was, however, not isolated during this study, but two *Cryptococcus* species of opportunistic importance, *Cryptococcus albidus* and *Cryptococcus randhawai* were detected. Of the two, *C. albidus* is of significance because it has also been found to occasionally cause moderate-to-severe meningitis, in human patients with compromised immunity due to HIV infection, cancer chemotherapy, metabolic immunosuppression, etc. [15,16].

*Fusarium* spp. are usually important for their role in the production of mycotoxins and only *F. solani* and *F. oxysporum* have been implicated in human diseases such as disseminated infections, keratitis, skin, and nail infections [12]. *F. polyphialidicum*, which was detected in this study, is only known as a plant pathogen [17].

*Rhodotorula* species have emerged as opportunistic pathogens that have the ability to colonize and infect susceptible patients. Among the *Rhodotorula* species, *Rhodotorula mucilaginosa* and *Rhodotorula kratochvilovae* were detected in our study. Of the two, only *R. mucilaginosa* is commonly isolated from foods and beverages and is a known opportunistic pathogen causing *Rhodotorula* fungemia associated with central catheters in patients with hematologic malignancies [18].

Apart from the fungi mentioned above, the other fungi detected in our study, namely, *Graphium dubautiae*, *Myrmecridium schulzeri*, *Naganishia albida*, *Paecilomyces lilacinus*, and *Zygopleurage zygospora* were not found to be of any human health significance despite an extensive literature search. However, further investigations may be required to completely establish a low risk.

## Conclusion

This study found a number of opportunistic fungi which should arouse public health interest as humans share a contaminated environment with pigeons. Fungi that are opportunistic to the immunosuppressed and the old are especially of interest and should stimulate interest of stakeholders. Further and more specific studies are therefore recommended in order to establish the full extent of the risks the pigeons are responsible for.

## Authors’ Contributions

TR performed the experiments and wrote the first draft, NL provided the analysis tools and analyzed the data, and MS conceived and designed the experiments, provided reagents, materials, and wrote the final paper. All authors read and approved the final manuscript.
